# Neighborhood-Level Inequalities in Dental Care of Adolescents and Young Adults in Southwestern Ontario

**DOI:** 10.3390/children9020183

**Published:** 2022-02-01

**Authors:** Naima Abouseta, Noha Gomaa, S. Jeffrey Dixon, Sharat Chandra Pani

**Affiliations:** 1 Department of Medical Biophysics, Schulich School of Medicine & Dentistry, University of Western Ontario, London, ON N6A 5C1, Canada; nabouset@uwo.ca; 2 Oral Diagnostic Sciences, Schulich School of Medicine & Dentistry, University of Western Ontario, London, ON N6A 5C1, Canada; noha.gomaa@schulich.uwo.ca; 3 Epidemiology and Biostatistics, Schulich School of Medicine & Dentistry, University of Western Ontario, London, ON N6G 2M1, Canada; 4 Children’s Health Research Institute, Lawson Health Research Institute, London, ON N6C 2R5, Canada; 5 Department of Physiology and Pharmacology, Schulich School of Medicine & Dentistry, University of Western Ontario, London, ON N6A 5C1, Canada; jeff.dixon@schulich.uwo.ca; 6 Pediatric Dentistry, Schulich School of Medicine & Dentistry, University of Western Ontario, London, ON N6A 5C1, Canada

**Keywords:** dental treatment outcomes, geographic information system (GIS), adolescent oral health, young adults, oral health inequality, cost of dental care

## Abstract

We examined whether the association of neighborhood-level socioeconomic status (SES) with the cost of dental care and dental care outcomes differs between adolescents and young adults. A total of 2915 patient records were split into two groups: adolescents (15 to 17 years of age) and young adults (18 to 24 years of age). Three dental care outcomes—routine oral evaluation (OEV-CH-A), utilization of preventive services (PRV-CH-A), and dental treatment services (TRT-CH-A)—were determined according to the Dental Quality Alliance (DQA) criteria. Associations of neighborhood SES and other sociodemographic variables with dental care outcomes and the cost of dental care were assessed using binary logistic and univariate linear regression models, respectively. Young adults had significantly lower PRV-CH-A and higher TRT-CH-A scores when compared to adolescents. We observed a significant negative association between TRT-CH-A and median household income in both adolescents and young adults. Utilization of dental treatment services was positively associated with the cost of care in both age groups, whereas utilization of preventive services was inversely associated with the cost of care in young adults, but not in adolescents. Neighborhood-level income was inversely associated with increased TRT-CH-A in both young adults and adolescents. In summary, young adults showed significantly worse preventive and treatment outcomes when compared to adolescents. Moreover, individuals from neighborhoods with a lower household income showed a significantly higher cost of dental care, yet worse treatment outcomes.

## 1. Introduction

The transition from adolescence to adulthood is marked by several changes that could impact the health and well-being of individuals [1]. The oral health of adolescents and young adults has recently begun to receive attention in the literature. Although dental caries is the most common health problem for adolescents [2,3] research suggests that adolescents are also at a risk of other oral diseases, such as traumatic dental injuries and periodontal diseases [2]. Individuals who suffer from higher levels of dental caries in childhood may be also more prone to developing dental problems in adulthood, a fact that supports the need to study dental caries and dental care outcomes as children transition to adolescence and adulthood [4].

In addition to being influenced by genetics and health behaviors, several social and economic changes that occur as one transitions from adolescence to adulthood can also impact oral health [5,6]. For example, in Ontario, children under the age of 18 years who are from low-income households are eligible for government-funded dental care through the Healthy Smiles Ontario (HSO) program [7], which essentially means that adolescents (15 to 17 years of age) from low-income households can access dental care through this program, whereas young adults (18 to 24 years of age) cannot. 

Worldwide, the cost of treatment of oral diseases, whether paid for by public or private insurance or by the patient, can be a barrier to accessing dental care for those with limited financial means [8]. Canada’s oral health care system is largely privatized; approximately 60% of expenditures are financed by private insurance, such as employer or individual plans, and 35% are paid directly by the patient. Theoretical models for caries risk prediction, based on social and demographic variables, show that these barriers are borne disproportionately by socially disadvantaged populations [9,10]. Income-related inequalities persist even when patients have dental insurance coverage and good oral hygiene practices [11]. Canadians living in households with lower incomes are less likely to visit a dentist, with the proportion reaching 49.6% amongst those with low income and without insurance [12,13]. The association between socioeconomic status (SES) and oral health has been widely studied, however there has been little research on how differences in SES influence oral health in specific age groups. Although there has been some research using advanced methods to explore oral health inequality in Canada, there is little information on the magnitude of inequalities in adolescents and young adults, or on the possible impact of the loss of government-funded programs at 18 years of age. The cost of dental care is often a reflection of the treatment sought, and billing data has been shown to be an indicator of both access to dental care and the quality of dental care outcomes [14].

Although advances in technology have led to rapid progress in the field of data mining [14,15], it has not been used widely to interrogate Canadian oral health billing databases. Protocols for collecting electronic dental billing data have been proposed by the Dental Quality Alliance (DQA) and have been used successfully to measure the quality of dental care received [15,16]. Neighborhood-level sociodemographic data in Canada are available at the level of forward sortation area (FSA) postal codes [17], which have been used by both diabetes and cancer researchers as a measure of neighborhood-level socioeconomic status [18,19]. There is some data on the mapping of dental caries according to postal codes in children [20], and the relationship between dental billing data and neighborhood-level sociodemographic variables among Canadian children has recently been explored [21]. The present study examined whether the association of neighborhood-level socioeconomic status (SES) with the cost of dental care and dental care outcomes differs between adolescents and young adults. 

## 2. Methodology

### 2.1. Ethics Approval

This research was approved by the Health Sciences Research Ethics Board (HSREB) at the University of Western Ontario (2020-115567-37532) and the use of secondary data was conducted within the principles and guidelines of the Canadian Tri-Council Policy Statement on Ethical Conduct for Research Involving Humans [22].

### 2.2. Screening of Patient Records

The present study utilized secondary billing data from the records of patients visiting the dental clinics of the Schulich School of Medicine & Dentistry at the University of Western Ontario in London, Ontario. Electronic dental records of patients aged 15–24 years at the time of the last dental visit were screened. Patients who had at least one additional treatment code within a 180-day period of the first recorded visit for each year studied were included in the study according to the protocol set by the DQA to ensure that there is no skew or bias [16,23]. Only FSA codes with at least 20 patients were included in the study. A total of 2915 patients met the inclusion criteria and were split into two age groups: adolescents (15 to 17 years of age) and young adults (18 to 24 years of age).

### 2.3. Variables

Dependent variables: Dental care outcomes were operationalized using the following DQA criteria: (a) OEV-CH-A, whether a patient had received a comprehensive or periodic oral evaluation within the year studied; this included adolescents and young adults who had at least one scheduled oral examination in a year, which included a complete exam, a recall oral exam, or an oral surgery specific exam; (b) PRV-CH-A, whether a patient received at least one preventive measure within the year studied; this included adolescents and young adults who had received either pit and fissure sealants, oral prophylaxis, or scaling; and (c) TRT-CH-A, whether a patient received at least one treatment service within the year studied; this included adolescents and young adults who had received endodontic or restorative treatment, or had a tooth extracted. 

These variables were operationalized as a binary (yes or no). The cost of dental care was recorded as per the Ontario Dental Association (ODA) fee guide [23] as well as the subsidized fee charged by the dental school. 

Independent variables: Neighborhood-level SES was obtained through anonymized sociodemographic data and the first three digits of the patient’s postal code, which were retrieved from the records. These were matched to data from Statistics Canada, which are stored by postal code and are readily available online [17]. The first three characters of the postal code are referred to as the forward sortation area (FSA) code, which allows for the collection of geographic data while maintaining the confidentiality of the identity of individuals. Neighborhood-level variables included median household income, percentage of the population with less than secondary school education, percentage of the population whose language spoken at home was not an official language in Canada (i.e., neither English or French), and the percentage of the population that had lived in Canada for less than 10 years.

### 2.4. Data Coding and Mapping

Coding was performed using criteria and methods that have been previously published [21]. Neighborhood-level dental care outcomes were geovisualized separately for each age group using the geographic information system software ArcGIS 10.8.1 (ESRI Canada, Toronto, ON, Canada). The data on the neighborhood-level independent variables previously mentioned were downloaded for each FSA code from the Statistics Canada database. The DQA variables for each patient were entered into the software according to their FSA code. Viable data (>20 individuals) was obtained from 17 FSA codes in the metropolitan area of London, Ontario, Canada. The entered data were used to create maps to geovisualize both neighborhood-level sociodemographic and dental care outcome variables. To facilitate visualization, maps were generated only for FSA codes (*n* = 14) that fell within the city limits of London, Ontario.

### 2.5. Statistical Analyses 

First, descriptive statistics were applied. We used the Student’s *t*-test to assess differences in study sample characteristics and the cost of dental care between the two age groups. The three dental care outcomes were then compared between the two age groups using the Mann–Whitney U test. We constructed three separate binary logistic regression models for each of the age groups to assess the association of OEV-CH-A, PRV-CH-A, and TRT-CH-A as dependent variables with neighborhood-level SES independent variables. 

The sample was modelled according to FSA code using the following as covariates: median household income, percentage of the population with less than secondary school education, percentage of the population speaking a non-official language at home, and the percentage of the population that had lived in Canada for less than 10 years. The association between these neighborhood-level sociodemographic variables and individual cost of dental care was assessed using univariate linear regression models. 

## 3. Results

A total of 2915 patients (1640 males, 1200 females, and 75 preferred not to disclose gender) from a total of 17 FSA codes in the London metropolitan area met the inclusion criteria. The mean age for patients in this sample was 19.7 years (SD ± 2.9 years) (Table 1). The mean cost of dental care was CAD 208 (SD ± 251) using the subsidized rates for the dental school and CAD 433 (SD ± 526) using the recommended provincial fee guide (Table 1). The cost of dental care was significantly greater for young adults (ODA fees CAD 512, SD ± 576) compared to adolescents (ODA fees CAD 194, SD ± 179) (*t* = −21.111, *p* < 0.001). 

When dental care outcomes were compared between the two age groups, it was observed that, although most of the individuals in both age groups received a routine dental examination (OEV-CH-A), the proportion was significantly greater in young adults (67.9%) when compared to adolescents (56.7%). Significantly more adolescents received preventive dental services (PRV-CH-A) (30.9%) compared young adults (18.6%). The Mann–Whitney U test found these differences to be significant for routine oral evaluation (*p* < 0.001), preventive services (*p* < 0.001), and treatment services (*p* < 0.001) (Table 2). 

**Figure 1:**
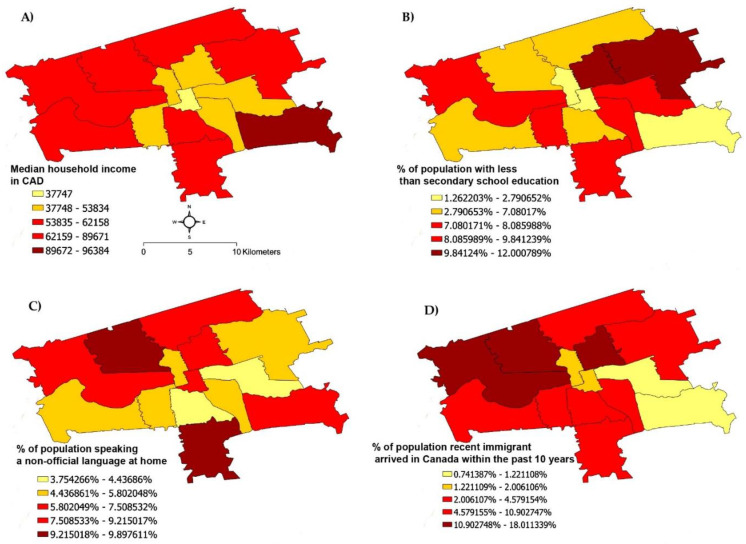
Geographic distribution of selected sociodemographic variables with the 14 FSA codes that lie within the City of London, Ontario, Canada: (**A**) median household income, (**B**) percentage of the population with less than secondary school education, (**C**) percentage of the population speaking a non-official language at home, and (**D**) percentage of the population who are recent immigrants (arrived in Canada in the past 10 years). Maps were created using data from Statistics Canada [17], with each outlined area representing a single FSA code. Distance scale in A applies to all panels.

The City of London has been recorded as having a wide range of sociodemographic groups [17]. The following neighborhood-level sociodemographic variables were geovisualized and separated according to FSA code boundaries: family’s median income, percentage of the population with less than secondary school education, speaking a non-official language at home, and those who had arrived in Canada within the past 10 years. The resultant maps showed distinct boundaries in terms of the four sociodemographic variables visualized, suggesting that visualization of the dental care outcome variables at an FSA level was feasible (Figure 1). Furthermore, the geographic distributions of the four chosen sociodemographic variables were distinct, suggesting the need to examine each as a separate variable in a regression model.

**Figure 2:**
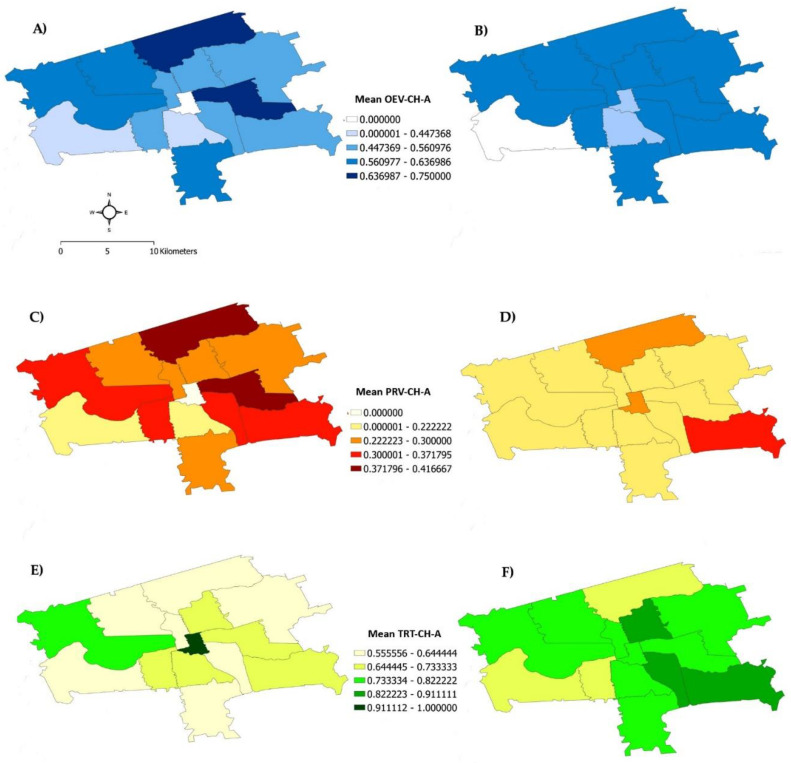
Geographic distribution of dental care outcome variables: (**A**,**B**) routine oral evaluation (OEV-CH-A), (**C**,**D**) preventive services (PRV-CH-A), and (**E**,**F**) dental treatment services (TRT-CH-A). Data for adolescents are shown in (**A**,**C**,**E**). Corresponding data for young adults are shown in (**B**,**D**,**F**). Numerical values represent mean DQA scores on a scale from 0 to 1. Distance scale in A applies to all panels. To facilitate visualization, the maps include only FSA codes (*n* = 14) that fall within the city limits of London, Ontario.

When the dental care outcomes were geovisualized using the same 14 FSA codes, distinct patterns of care were seen. Comparing data for adolescents and young adults, it was observed that there was an overall increase in OEV-CH-A scores in young adults (Figure 2A,B). However, this increase was not uniform, with some FSA codes showing an increase in OEV-CH-A (darkening in the color of the FSA code) and others showing a decrease (lightening in the color of the FSA code), suggesting potential geographic inequalities in access to routine dental care. Moreover, some areas showed a decrease in PRV-CH-A scores (lightening in the color of the FSA code) and an increase in the TRT-CH-A score (darkening in the color of the FSA code) in young adults when compared to adolescents (Figure 2). 

Logistic regression models showed that median household income was not significantly associated with the OEV-CHA in the population studied. However, it was observed that patients from FSA codes with a greater percentage of the population speaking a non- official language at home or recently immigrated to Canada were more likely to visit the dental clinic of the school (Table 3). There was no significant association between PRV-CH-A and median household income in adolescents (OR = 1.0, 95% CI 0.8–1.1), but young adults from higher income families were more likely to receive preventive dental care than those from lower income families (OR = 1.2, 95% CI 1.0–1.3). The model also showed significant inverse associations between median neighborhood-level household income and dental treatment services in both age groups, suggesting that families with a higher median income had a lower risk of dental treatment (OR = 0.9) (Table 3).

OEV-CH-A was associated with a significant increase in the cost of care in adolescents (OR = 1.3, 95% CI 1.0–1.6), but not in young adults (OR = 0.9, 95% CI 0.8–1.0) (Table 4). PRV-CH-A was associated with a significant decrease in the cost of dental care in young adults (OR = 0.7, 95% CI 0.6–0.8), but not in adolescents (Table 4). TRT-CH-A was associated with a significant increase in the cost of care in both adolescents (OR = 3.2, 95% CI 2.1–4.9) and young adults (OR = 18.4, 95% CI 10.8–31.6) (Table 4).

Linear regression models with the cost of dental care as the dependent variable showed that the median household income was inversely associated with the cost of dental care, a finding that was significant in young adults (*p* < 0.001), but not in adolescents (*p* = 0.161) (Table 5). The percentage of the population with less than secondary school education was significantly associated with the cost of care among adolescents (*p* = 0.044), but not young adults (*p* = 0.200). There was a significant positive association between the cost of dental care and the percentage of the population that spoke a non-official language in both age groups.

## 4. Discussion

Andersen’s behavioral model suggests that healthcare (including dental care) is dependent on both individual and societal elements [24,25]. While much of the previous work on quality of dental care has focused on the individual [15,16,26], it is only recently that attempts have been made to visualize societal data at a geographic level [20,21]. The transition from adolescence to adulthood is affected by a number of personal conditions, such as beliefs or attitudes, SES, and societal conditions, such as marginalization, gender, and race or ethnicity, which can facilitate or impede the transition [27,28]. The present study sought to visualize the factors influencing dental care outcomes at a neighborhood level, and the differences in these outcomes between adolescents and young adults.

Our study used a cross-sectional design, assessing dental utilization through the data mining of electronic health records and billing data. This has been demonstrated to be an accurate indication of both dental caries risk and access to dental care [26]. We utilized data from a subsidized dental clinic, to examine the impact of transitioning from adolescence to young adulthood in a population where access to dental care was available. The fact that routine oral health visits increased among young adults when compared to adolescents suggests that the access to dental care in this population did not decrease after the age of 18 years. 

Access to dental care alone is not an accurate marker of the quality of dental care received, with studies showing that even when individuals have access to dental care, social and economic factors can influence the quality of care received [28,29,30]. Our study utilized three of these measures to quantify access to routine dental care (OEV-CH-A), preventive dental services (PRV-CH-A), and treatment procedures (TRT-CH-A). The results suggest a higher OEV-CH-A among young adults when compared to adolescents, however, this must be viewed whilst keeping in mind the fact that the DQA requires a minimum of two dental visits in a year for the individual to be included in the study [15]. Despite having higher numbers of individuals with access to care, young adults had a significantly lower number of visits for preventive care and a significantly greater number of visits for dental treatment. This, along with the significantly greater cost of dental care among the young adults, suggests that the young adults had poorer dental care outcomes when compared to adolescents. This finding is in keeping with the pressures faced by young adults as they transition from living with their parents to living independently and making independent life choices [31]. As young adults become more independent from parental influences, they are likely to have increased responsibility for their own oral health and dental visits [31]. The fact that there was a significant change in the 18–24-year age group is in keeping with the findings in France that resulted in creation of the M’T Dents program that extends pediatric oral health benefits up to the age of 24 years [30]. 

The average cost of dental care per patient from areas having a higher median household income was less than that of patients from areas with lower median household income. This was seen in both the 15–17-year age group as well as the 18–24-year age group, consistent with the findings of other studies showing that individuals from lower income families may end up spending more on dental care [32,33]. The findings are also in keeping with those of a similar study conducted on children below 15 years of age [21], suggesting that socioeconomic determinants of healthcare affect individuals across different age groups. 

It was observed that the presence of TRT-CH-A increased the cost of dental care, however, PRV-CH-A and OEV-CH-A did not. Furthermore, it was observed that, in the 18–24-year age group, there was a significant negative association between the cost of dental care and PRV-CH-A. This supports the argument made by previous studies that regular preventive dental care can reduce overall dental treatment costs [34,35]. The findings of our study are in keeping with the idea that this relationship occurs not only at an individual level but also at a neighborhood level. 

Our findings revealed that the family median income only became important after the age of 18 years. In the 18–24-year group, individuals from neighborhoods with a higher household income were significantly more likely to receive preventive services and significantly less likely to receive treatment services. This is in keeping with individual-level research among both adolescents [36] and adults [37]. The geovisualization of these variables also showed that the changes in dental care outcomes were more pronounced in some FSA codes when compared to others The results of this study show neighborhood-level discrepancies in dental care outcomes in both the age groups studied. This is in keeping with a previous study on children in the same population [21]. However, the limited number of FSA codes did not allow for the use of more powerful geographic regression models in this study. There may be several factors that influence these geographic variations (e.g., dentist population ratios, connectivity to the dental school, accessibility to healthcare in the neighborhood, etc.). However, there is little regional or province-wide data available on such factors, and this may be an interesting area for future research.

The results of the study need to be viewed whilst keeping in mind certain limitations. This study only examined individuals visiting the subsidized clinics of a dental school and therefore might not be representative of the entire population. Furthermore, only 17 codes were included in the study, which means that it was not possible to apply more rigorous neighborhood-level regression modelling, such as the ordinary least squares (OLS) model. There is a need for studies using a larger sample size of FSA codes and including private clinics as a source of data to explore the different socioeconomic and demographic variables documented through census data in Canada.

## 5. Conclusions

The results of this study show that young adults have significantly poorer preventive and treatment outcomes when compared to adolescents. Individuals from neighborhoods with lower household incomes had significantly greater costs of dental care and poorer dental care outcomes.

## Figures and Tables

**Table 1:** Characteristics of study sample.

Age Group ^a^	Variable	Sex	Mean	SD	*t* *	Sig **
15–17 years (*n* = 817)	Age (years)	Male	16.00	0.84	0.324	0.572
		Female	15.98	0.83		
	ODA Fees (CAD) ^b^	Male	194.96	201.09	0.115	0.094
		Female	193.30	151.59		
	Subsidized Fees (CAD)	Male	76.48	95.80	1.600	<0.001 **
		Female	66.61	46.00		
18–24 years (*n* = 2023)	Age (years)	Male	21.27	2.00	2.294	0.508
		Female	21.07	1.99		
	ODA Fees (CAD) ^b^	Male	513.73	598.75	0.165	0.773
		Female	509.21	545.54		
	Subsidized Fees (CAD)	Male	253.60	280.49	0.222	0.983
		Female	250.76	260.40		

* Calculated using the independent *t* test. ** Indicates significant difference between sexes. ^a^ Numbers do not include the 75 individuals who preferred not to disclose their gender. ^b^ Calculated based on those participants who paid for services that were billable using an ODA fee code (*n =* 624 for 15–17 years of age; *n* = 1888 for 18–24 years of age).

**Table 2:** Comparison of dental care outcomes between the two age groups.

Oral Variables	Age Group	DQA Outcome	Observations (*n* = 2915)	Proportion	Sig *
Routine oral evaluation (OEV-CH-A)	15–17 years	Absent	360	43.3%	<0.001
		Present	472	56.7%	
	18–24 years	Absent	669	32.1%	
		Present	1414	67.9%	
Preventive services (PRV-CH-A)	15–17 years	Absent	575	69.1%	<0.001
		Present	257	30.9%	
	18–24 years	Absent	1695	81.4%	
		Present	388	18.6%	
Dental treatment services (TRT-CH-A)	15–17 years	Absent	302	36.3%	<0.001
		Present	530	63.7%	
	18–24 years	Absent	481	23.1%	
		Present	1602	76.9%	

* Indicates significant difference between age groups, calculated using the Mann–Whitney U Test.

**Table 3:** Binary logistic regression models for the associations between dental care outcomes and neighborhood-level demographic variables.

	Neighborhood-Level Variables ^a^	Oral Evaluation ^1^ (OEV-CH-A) OR (95% CI)	Preventive Services ^2^ (PRV-CH-A) OR (95% CI)	Dental Treatment Services ^3^ (TRT-CH-A) OR (95% CI)
15–17 years	Median household income	1.1 (0.9, 1.2)	1.0 (0.8, 1.1)	0.9 (0.7, 1.0)
	% of population with less than secondary school education	0.9 (0.8, 1.0)	1.1 (0.9, 1.2)	1.0 (0.8, 1.1)
	% of population speaking a non-official language at home	1.2 (1.1, 1.4)	1.0 (0.8, 1.1)	1.0 (0.9, 1.2)
	% of population recent immigrant arrived in Canada within the past 10 years	1.2 (1.0, 1.4)	1.0 (0.9, 1.2)	1.0 (0.9, 1.2)
18–24 years	Median household income	0.9 (0.9, 1.0)	1.2 (1.0, 1.3)	0.9 (0.8, 0.9)
	% of population with less than secondary school education	0.9 (0.8, 1.0)	1.0 (0.9, 1.1)	1.1 (1.0, 1.2)
	% of population speaking a non-official language at home	1.1 (1.0, 1.2)	1.0 (0.9, 1.2)	1.0 (0.9, 1.1)
	% of population recent immigrant arrived in Canada within the past 10 years	1.1 (1.0, 1.2)	1.0 (0.9, 1.1)	1.0 (0.9, 1.2)

^a^ Calculated using average FSA code level data from the Statistics Canada Database, ^1^ calculated using binomial logistic regression with OEV-CH-A as dependent variable, ^2^ calculated using binomial logistic regression with PRV-CH-A as dependent variable, and ^3^ calculated using binomial logistic regression with TRT-CH-A as dependent variable.

**Table 4:** Binary logistic regression models for the associations between cost of care and dental care outcomes.

Dental Outcome Measure	Age Group	OR (95% CI)	Sig
Routine oral evaluation (OEV-CH-A)	15–17 years	1.3 (1.0, 1.6)	0.029 *
	18–24 years	0.9 (0.8, 1.0)	0.128
Preventive services (PRV-CH-A)	15–17 years	1.1 (0.9, 1.3)	0.241
	18–24 years	0.7 (0.6, 0.8)	0.001 *
Dental treatment services (TRT-CH-A)	15–17 years	3.2 (2.1, 4.9)	0.001 *
	18–24 years	18.4 (10.8, 31.6)	0.001 *

* Indicates significant association between cost of care and indicated dental care outcomes.

**Table 5:** The association of neighborhood-level demographic variables with cost of dental care.

Age Group	Neighborhood-Level Variables ^a^	B *	Sig	95% CI
15–17 years	Median household income (CAD)	−0.058	0.161	(−0.140, 0.023)
	Percentage of population with less than secondary school education	0.105	0.044 **	(0.003, 0.207)
	Percentage of population speaking a non-official language	0.160	0.031 **	(0.014, 0.306)
	Percentage of population who arrived in Canada within the past 10 years	−0.116	0.075	(−0.243, 0.012)
18–24 years	Median household income (CAD)	−0.087	<0.001 **	(−0.134, −0.041)
	Percentage of population with less than secondary school education	0.035	0.200	(−0.018, 0.088)
	Percentage of population speaking a non-official language	0.099	0.013 **	(0.021, 0.177)
	Percentage of population who arrived in Canada within the past 10 years	−0.016	0.668	(−0.087, 0.056)

^a^ Calculated using average FSA code level data from the Statistics Canada Database. * Calculated using linear regression model with cost of care as the dependent variable. ** Indicates significant association.

## Data Availability

The data will be provided upon reasonable request to the authors.
